# *PLOS Biology* 2014 Reviewer Thank You

**DOI:** 10.1371/journal.pbio.1002101

**Published:** 2015-02-27

The Public Library of Science (PLOS) and the *PLOS Biology* editorial team would like to warmly thank all those individuals who participated in the peer review process this past year at *PLOS Biology*. During 2014, over 1,250 reviewers from around the world helped evaluate articles submitted to the journal.

The names of our 2014 reviewers are listed in S1 Reviewer List. We are grateful to all our reviewers for sharing your expertise with *PLOS Biology* authors, and for helping us to publish a highly valued open access journal.

## Supporting Information

S1 Reviewer List Alphabetical list of 2014 *PLOS Biology* reviewers. (PDF)

## References

[1] (2015) *PLOS Computational Biology* 2014 Reviewer Thank You. PLoS Comput Biol 11(2): e1004184 10.1371/journal.pcbi.1004184

[2] (2015) *PLOS Genetics* 2014 Reviewer Thank You. PLoS Gen 11(2): e1005070 10.1371/journal.pgen.1005070

[3] (2015) *PLOS Medicine* 2014 Reviewer Thank You. PLoS Med 12(2): e1001806 10.1371/journal.pmed.1001806

[4] (2015) *PLOS Neglected Tropical Diseases* 2014 Reviewer Thank You. PLoS Negl Trop Dis 9(2): e0003621 10.1371/journal.pntd.0003621

[5] (2015) *PLOS ONE* 2014 Reviewer Thank You. PLoS ONE 10(2): e0121093 10.1371/journal.pone.0121093

[6] (2015) *PLOS Pathogens* 2014 Reviewer Thank You. PLoS Pathog 11(2): e1004748 10.1371/journal.ppat.1004748

