# Supplementary material for: PLOS Biology 2014 Reviewer Thank You
Source: PLoS Biol. 2015 Feb 27;13(2):e1002101. doi: 10.1371/journal.pbio.1002101 (PMC4344249; doi:10.1371/journal.pbio.1002101)
Supplement: S1 Reviewer List — (PDF) [file pbio.1002101.s001.pdf]

PLOS Biology would like to thank all those who reviewed on behalf of the journal in 2014:

Alejandro Aballay
Asa Abeliovich
Arhat Abzhanov
Martin Ackermann
Christoph Adami
Karen Adelman
Sankar Adhya
Markus Affolter
Kiyokazu Agata
Aneil Agrawal
Nadav Ahituv
Chul Ahn
Michael Alfaro
Juan Alfonzo
Robin Ali
Benjamin Allen
Stefano Allesina
Uri Alon
Patrick Aloy
David Amaral
Angelika Amon
Kathryn Anderson
Adam Anderson
Jill Anderson
Raul Andino
Robert Anholt
A Aricescu
Robert Arkowitz
Luc Arnal
Gustavo Arrizabalaga
Alberto Ascherio
Gregory Ashby
Mark Ashe
John Assad
Hellmut Augustin
Johan Auwerx
Bruno Averbeck
Edward Awh
Segolene Ayme
Wolfgang Bach
Andreas Bachmair
Doris Bachtrog
Roland Baddeley
Jaideep Bains
David Baker
Robert Baldwin
Frances Balkwill
Steven Ball
Andre Ballesteros-Tato
David Baltrus
Pavel Baranov
Daniel Barbash
Naama Barkai
Timothy Barraclough
Yves Barral
Ben Barres
Amy Barrios
Jiri Bartek
David Bartel
Nick Barton
Michelle Barton
Allan Basbaum
Hassan Bassem
Jochen Bassler
Gillian Bates
Martin Bauer
Diana Bautista
John Beaver
Lino Becerra
Oren Becher
Moriah Beck
Kate Beckingham
Mike Begon
Konstantinos Beis
Hugo Bellen
Leonardo Belluscio
Monsef Benkirane
Richard Bennett
Elena Bennett
Roger Benson
Alberto Bernacchia
Anne Bertolotti
Joseph Besharse
Leo Beukeboom
Upinder Bhalla
Margherita Bignami
Marom Bikson
Emanuele Biondi
Douglas Bishop
Michael Blackman
Kim Blackwell
Isobel Blake
Cedric Blanpain
Mark Blaxter
Maryse Block
Brenda Bloodgood
Theodora Bloom
Martin Blum

Matthew Bogyo
Johan Bolhuis
Kirsten Bomblies
Russell Bonduriansky
Vincent Bonin
Aletta Bonn
Dominique Bonnet
Henry Boom
Erie Boorman
Mike Boots
David Borchelt
Justin Borevitz
Jean-Paul Borg
Erich Bornberg-Bauer
David Borsook
Anne-Gaelle Borycki
Giovanni Bosco
Mark Bothwell
Henri-Marc Bourbon
James Bourne
Isabelle Boutron
Michael Boutros
Derek Bowie
Jon Boyle
Oliver Braddick
Peter Bradley
Andre Brandli
Thomas Braun
Michael Brecht
Rachel Brem
Joshua Brickman
Constance Brinckerhoff
Tim Brodribb
John Brookfield
Thomas Brooks
Charles Brooks III
Christel Brou
Martina Brueckner
Michael Bruford
Anne Brunet
Tania Bubela
Gershon Buchsbaum
Lauren Buckley
Ralf Buckley
Angus Buckling
Sarah Budischak
Bernd Bukau
Daniel Bullock
James Bullock
Martha Bulyk
Stephen Buratowski
Boudewijn Burgering
Wolfgang Busch
Jerry Busemeyer
Roger Butlin
Roberto Cabeza
Ken Cadigan
Stefano Calza
A. Malcolm Campbell
Ray Campbell
Judith Campisi
Isaac Cann
Jessica Cantlon
Rut Carballido-Lopez
Charlie Carlson
Kristian Carlson
Ginger Carney
Vern Carruthers
Water Carson
Vivien Casagrande
Christian Casanova
James Castelli-Gair Hombría
Jamie Cate
William Catterall
Maurice Chacron
Richard Chadwick
Remy Chait
Douglas Chalker
Ian Chambers
Chris Chambers
Andrew Chan
Jonah Chan
Deborah Charlesworth
Brian Charlesworth
Catherine Charneski
Frederic Charron
Julia Chekanova
Peter Cherepanov
Mehdi Cherif
Roberto Chiesa
Takahiro Chihara
Andrew Chisholm
Ajay Chitnis
Lars Chittka
Giltsu Choi
Yves Choquet
Gerardo Chowell
Jonathan Chubb
Jerold Chun
Cheng-Ming Chuong

Gul Civelekoglu-Scholey
Michael Clarke
Stephen Cobbold
Cynthia-Lou Coleman
Nansi Colley
Harry Collins
Rita Colwell
Joan Conaway
Maria Concetta Morrone
Cathy Conrad
Barbara Conradt
Daniel Constam
Peter Cook
Erik Cook
Kim Cooper
Anita Corbett
Matthew Cordes
Leah Cowen
Keith Crandall
Neil Crickmore
Lee Cronin
Jody Culham
Paul Cullen
Asher Cutter
Ira Daar
Matteo Dal Peraro
Ross Dalbey
Marc Dalod
John Dame
Aniruddha Das
Jeremy Dasen
Mehul Dattani
Richard Davidson
Jamie Davies
Jonathan Davies
Graeme Davis
Alice Davy
Troy Day
Alexis De Angeli
Xavier De Bolle
Mario De Bono
Jose De Celis
Floris De Lange
Hans De Longh
Sylvia De Pater
johan De Rooij
Abbe De Vallejo
Lieven De Veylder
Peter De Weerd
Dominique Debanne
Flo Débarre
Eric Deeds
Jason Delborne
Dean Della Penna
Robert Dempski
R. Ford Denison
Eric Denkers
Erik Dent
Brian Derby
Catherine deRivera
Rik Derynck
Robert Deschenes
Raymond Deshaies
Robert Desimone
Patricia Di Lorenzo
Marian DiFiglia
Mingzhou Ding
Marc Dionne
Jochen Ditterich
Andy Dobson
Michael Doebeli
Xinnian Dong
Xinzhong Dong
Maria Donoghue
Nico Dosenbach
John Drake
David Drew
Iain Drummond
D. Drummond
George Drusano
J. Emmett Duffy
Jeffrey Dukes
Sophie Dumont
Ray Dunn
Anindya Dutta
Steve Dworkin
Daniel Eberl
Dieter Ebert
Karen Echeverri
Tassos Economou
Bruce Edgar
Virginia Edgcomb
Katrina Edwards
Jonathan Eggenschwiler
Barbara Ehrlich
A.E. Eiben
Howard Eichenbaum
Karl Ekwall
Ronald Ellis
Georgina Ellison

Amanda Ellison
Hana El-Samad
Ben Emery
Drew Endy
Lynn Enquist
Joel Ernst
Mark Estelle
Jay Evans
Jonathan Ewbank
Damien Fair
Robert Fairclough
Jin Fan
Bentley Fane
Christopher Fasano
Justin Fay
Martin Fenner
Brock Fenton
Stuart, Ferguson
Liz Ferguson
Neil Ferguson
Michael Ferns
Matthew Ferrari
Charles ffrench-Constant
Mark Field
Scott Filler
Urs Fischer
Wolfgang Fischle
John Flanagan
Ulf-Ingo Flügge
Alfredo Fontanini
Beatriz Fontoura
Kevin Fox
Jose Manuel Fragoso
Stephanie Fraley
Steven Frank
F. Chris Franklin
Mathias Franz
Christophe Fraser
Paul Freemont
Winrich Freiwald
Pascal Fries
Robert Froemke
Judith Frydman
Shigetomo Fukuhara
Barbara Funnell
Frédéric Gachon
Matt Gage
Fred Gage
Pascal Gagneux
Jean-Michel Gaillard
Niels Galjart
Jennifer Galovich
Nicolas Galtier
Manoj Gambhir
Edward Game
Sylvain Gandon
Laurent Gapin
Olga Garaschuk
K. Christopher Garcia
Bertrand Garcia-Moreno
Richard Gardner
Susan Gasser
Eric Gaucher
Sergey Gavrilets
Peter Geigenberger
Nicole Gerardo
Jean-Marc Ghigo
Geoffrey Ghose
Partho Ghosh
Shubha Ghosh
Amato Giaccia
Wendy Gilbert
Neil Gilbert
Lilach Gilboa
Florent Ginhoux
Gonzalo Giribet
Amy Gladfelter
Nate Glasser
Dale Godfrey
Daniel Goldberg
Barbara Golden
James Goldenring
Daniel Goldman
Ananda Goldrath
Heather Goldsby
Erin Goley
Franz Goller
Eva Gonzalez-Suarez
Peter Goodfellow
Geoffrey Goodhill
David Goodsell
Roger Goody
Jeff Gore
Bruno Goud
Elizabeth Gould
Shannon Gourley
Richard Gourse
Elvire Gouze
Anthony Graham
Andrea Graham

Ian Graham
Sarah Grant
Michael Gray
Charles Gray
Giuseppe Graziano
Henry Greely
Rachel Green
Jeremy Green
William Green
Jean Greenberg
E. Peter Greenberg
Miriam Greenberg
David Gresham
Bernd groner
Jerome Gros
Piet Gros
Claudio Grosman
Lou Gross
Carol Gross
Wesley Grueber
Joseph Grzymski
Haihua Gu
Raphael Guerois
Calin Guet
Jeremy Gunawardena
Matthias Gunzer
Sunetra Gupta
David Gurwitz
Eric Haag
Melissa Haendel
Gordon Hager
Steve Hahn
Heather Hain
Georg Halder
James Hall
Benedikt Hallgrímsson
Bin Han
William Hancock
Kasper Hansen
Simon Hanslmayr
J. Marie Hardwick
Michael Harfoot
Iswar Hariharan
Luke Harmon
Richard Harvey
Kieran Harvey
Bassem Hassan
Zahra Hassani
Philip Hastings
Michael Häusser
David Haussler
Karl Havens
Adam Hayward
Zuhua He
Biyu He
Tyson Hedrick
Jonathan Heeney
Ruth Heidelberger
Yael Heifetz
Carl-Philipp Heisenberg
Joseph Heitman
Randolph Helfrich
Johannes Hell
Michael Hemann
Rene Hen
Linda Hendershot
Ian Henderson
Graeme Henderson
Alvan Hengge
John Hershey
Michael Hickey
Tetsuya Higashiyama
Dawn Higginson
Stephen High
Okihide Hikosaka
Allan Hildesheim
Caroline Hill
David Hillis
Frank Hirth
Chris Hittinger
Oliver Hobert
Ian Hodge
Rolf Hoekstra
Patrick Hogan
Kristin Hogquist
Scott Hollister
Anthony Holmes
Ben Holt
Anthony Holtmaat
Frank Hölzle
Karl-Peter Hopfner
Norbert Hornstein
Alan Horsager
David Howells
Bing Hu
Sui Huang
Zachary Huang
Kerwyn Huang
Timothy Hughes
Stephen Hughes

David Hughes
Christopher Hunter
Greg Hurst
Benjamin Hutchinson
Anna Huttenlocher
Marko Hyytiäinen
Dagmar Iber
Axel Imhof
Nicholas Ingolia
Gareth Inman
Grzegorz Ira
Robin Irvine
Alain Israel
Janet Iwasa
Elisa Izaurralde
William Ja
Marja Jäättelä
F. Rob Jackson
Steven Jacobsen
Allan Jacobson
Matthew Jacobson
Joël Janin
Eckhard Jankowsky
Lars Jansen
Christopher Janus
Carlos Jaramillo
Heinrich Jasper
Richard Jefferson
Ole Jensen
Stefan Jentsch
Jason Jessen
Walter Jetz
Jin Jiang
Francis Jiggins
Tian Jin
Peng Jin
Jack Johnson
Matthew Johnson
Welkin Johnson
Graham Johnson
Thomas Jongens
Ferenc Jordán
Lynn Jorde
Natalia Jura
Vesa Kaartinen
David Kadosh
Matt Kaeberlein
Henrik Kaessmann
Klaus Kaestner
Jonathan Kagan
Michael Kahana
Marko Kaksonen
Joanne Kamens
Yukiyasu Kamitani
Harm Kampinga
Jack Kaplan
Zaven Kaprielian
Robert Kaptein
Prakash Kara
Katrin Karbstein
Istvan Karsai
Christoph Kayser
Amy Keating
Nancy Kedersha
Matt Keeling
Susan Kelly
Birgit Kemmerling
Kenneth Kemphues
Jennifer Kennell
Steven Kennerley
John Kenney
Jeffrey Kerby
Kamal Khanna
Thomas Kidd
John Kim
David Kimelman
Jonathan Kimmelman
Kayla King
Kirst King-Jones
Frank Kirchhoff
David Kirchman
Thomas Kirkwood
Alfredo Kirkwood
Marc Kirschner
Gretchen Kiser
Allon Klein
Daniel Kliebenstein
Richard Kliman
Wolfgang Klimesch
Stefan Klumpp
Robert Knight
Rob Knight
David Knipe
Laura Knoll
Barbara Knowlton
Elisabeth Knust
Guus Koch
Hiroki Koda
Barbara Koenig
Kyunghee Koh

PLOS | BIOLOGY

Hisato Kondoh
Genevieve Konopka
Ryszard Korona
Gautier Koscielny
Kenneth Kosik
Zoe Kourtzi
Ingo Kowarik
John Krakauer
Alexander Kraskov
Nina Kraus
Gabriel Kreiman
Andreas Kreiter
Skirmantas Kriaucionis
Dmitri Krioukov
Paul Kubes
Rolf Kuemmerli
Natalie Kuldell
Rohit Kulkarni
Dharshan Kumaran
Kazuhiko Kume
Zeb Kurth-Nelson
Michael Ladomery
Mato Lagator
Leon Lagnado
Anna-Liisa Laine
Christophe Lamaze
Arthur Lander
Robert Landick
Julia Lane
Jorg Langowski
Michael Lanzer
Hans Larsson
Joshua Lawler
Richard Lawrence
Karine Le Roch
Mark Leake
Terry Lechler
Youngsook Lee
Claire Legay
Laurent Lehmann
Ben Lehner
Ed Lein
Bernardo Lemos
Boris Lenhard
Pierre Léopold
François Leulier
Simon Levin
Gil Levkowitz
Kim Lewis
Randolph Lewis
Bing Li
Rong Li
Liheng Li
Min Li
Stephen Liberles
Phillip Lieberman
Wen-Hui Lien
Patrick Linder
Keith Lindsay
Keith Lindsey
Daniel Link
Klaus Linkenkaer-Hansen
Jan Liphardt
Tom Little
Dan Littman
Haoping Liu
Marta Llimargas
Thomas Lloyd
James Lloyd-Smith
Cristina Lo Celso
Laurence Loewe
Kyle Loh
Elizabeth Lonsdorf
Bingwei Lu
Rui Lu
Robert Lucas
Anneke Lucassen
Karolin Luger
Alberto Luini
Joen Luirink
Erik Lundquist
Bernhard Luscher
Hong Ma
Jian Ma
Andrew Maas
Frans Maathuis
Emiliano Macaluso
David Mackey
Malcolm Macleod
Judit Makara
Peter Makovicky
Leonard Maler
Harmit Malik
Hengye Man
Jim Manfredi
Raymond Mar
Gabriel Marais
Miguel Maravall
William Marcotte
Alberto Marina

Simone Marker
Chiara Marletto
Carsten Marr
Rogier Mars
Joseph Marsh
Tom Martin
Cathie Martin
Lynn Martin
Sergei Maslov
Bernard Mathey-Prevot
Ivan Matic
Daniel Matute
Roberto Mayor
Esteban Mazzoni
Kevin McCann
James McCaw
Dannel McCollum
Sheila McCormick
Michael McEachern
Jennifer McElwain
Brian McGill
Imelda Mcgonnell
Peter McHugh
J. Richard McIntosh
Jane McKeating
John McKinney
Marcel Mechali
Lin Mei
Johanna Meijer
Colin Meiklejohn
Karina Meiri
Markus Meissner
Jack Mellor
Lorne Mendell
Christophe Merceron
Hugo Merchant
Christien Merrifield
Jessica Metcalf
Matthew Meyerson
Stephen Michnick
Richard Michod
Daniel Mietchen
Samuel Miller
Jordan Miller
Baruch Minke
Tom Misteli
Jude Mitchell
James Mitchell
Thomas Mitchell-Olds
Shigeki Miyamoto
Mark Moasser
Kenneth Moberg
Amanda Moehring
Jeffrey Mogil
Denise Monack
Craig Montell
Arne Mooers
Lorna Moore
Camilo Mora
Francisco Moreno
Phil Morgan
Alexei Morozov
Levi Morran
Eric Moss
Daniel Mucida
Ralph Müller
Ulrich Müller
Ian Mulvany
Marcus Munafo
William Murphy
Andrew Murray
Cornelius Murre
Munitta Muthana
Michael Mwangi
Peter Nagy
Shinichi Nakagawa
Keiichi Namba
Inke Nathke
Kenneth Nealson
Matthew Neiditch
Martha Nelson
Ralph Nelson
Jennifer Nemhauser
Zoltán Neufeld
Elena Neumann
Dianne Newman
Hoang Nguyen
Christopher Nicchitta
Thomas Nichols
Timothy Nilsen
Ove Nilsson
Lee Niswander
Michael Nitabach
Hitoshi Niwa
Marcelo Nobrega
Toemme Noesselt
Peter Nonacs
MaryAnn Noonan
Mohamed Noor
Brian Nosek

Patrik Nosil
Reed Noss
Martin Nowak
Sergey Nuzhdin
Scott Oakes
Andrew Oberst
Michael O'Connor
John O'Doherty
Benjamin Ohlstein
Han Olff
Bruno Olshausen
Jill O'Reilly
Jessica O'Reilly
Merry Oursler
Kevin Padian
Michele Pagano
Tiago Paixao
Daniel Pak
Csaba Pál
Peter Palese
Ken Paller
Zhuo-Hua Pan
John Parkinson
Matthew Parsek
R. Jeroen Pasterkamp
Ardem patapoutian
Laszlo Patthy
Diane Paul
Walter Paulus
Norman Pavelka
Wojciech Pawlina
Laurence Pearl
Jean Peccoud
Jonathan Peelle
Stephan Peischl
Ari Pekka Mahonen
Mario Pende
Marta Perego
Sarah Perrett
Charles Perrings
Luiz Pessoa
Rasmus Petersen
Christian Petersen
Dmitri Petrov
Tatiana Petrova
Hervé Philippe
William Piel
Paul Pilch
Ramesh Pillai
Stuart Pimm
Lian Pin Koh
Heather Pinkett
Virginia Pitzer
Marysia Placzek
Matthias Platzer
Russell Poldrack
Thomas Pollard
David Pollock
Mu-Ming Poo
Martin Pool
Michael Posner
Hugh Possingham
Barbara Prainsack
Manu Prakash
Domenico Praticò
Alison Preston
Peter Prevelige
Vincent Prevot
Samantha Price
Nicholas Priebe
Victoria Prince
Andreas Prokop
Kay Prüfer
Jo Puglisi
Michael Purugganan
Bin-Zhi Qian
Alfredo Quinones-Hinojosa
Lars Råberg
Daniel Rabosky
Shahin Rafii
David Raichlen
Oliver Rando
John Rathjen
Scott Rauch
Mark Rausher
Andrew Read
Donald Ready
Venugopala Reddy Gonehal
A David Redish
Geraint Rees
Roland Regoes
David Reich
Dejian Ren
Susanne Renner
Max Reuter
Kim Rewitz
Sarah Rice
Kym Rice
Jeremy Rich
William Richardson

Bruce Riley
Steven Riley
Jasper Rine
Dagmar Ringe
Marylyn Ritchie
David Rizzo
Susan Roberts
Sonia Rocha
Ingo Roeder
Martijn Roelandse
Pieter Roelfsema
Henk Roelink
Tim Rohe
Jason Rohr
Jens Rolff
Edmund Rolls
Michael Rosbash
Michael Rose
Siegfried Roth
John Rouse
François Rouyer
Alex Rowe
Peter Roy
Jessica Royles
Denis Roze
David Rubinsztein
Stefan Rüdiger
Iñaki Ruiz-Trillo
James Russell
Edward Ruthazer
Guy Rutter
Bernardo Sabatini
Robert Sablowski
Lynn Sakai
Patricia Salinas
Rene Salinas
Leona Samson
Jürgen Sandkühler
Sanjay Sane
Susanna-Assunta Sansone
Casim Sarkar
Rashmi Sasidharan
Nathaniel Sawtell
Rebecca Saxe
Katja Schenke-Layland
Ben Scheres
Elmar Schiebel
Paul Schimmel
Larry Schlesinger
Christian Schlötterer
Karl Schmid
Martina Schmidt
Johanna Schmitt
David Schneider
Elad Schneidman
Danny Schnell
Gunnar schotta
Julian Schroeder
Oren Schuldiner
Maya Schuldiner
Gerhard Schütz
Ralph Schwarz
Luca Scorrano
Kristin Scott
Matthew Seaman
Sarah Seiter
Terrence Sejnowski
Posa Selkton
Scott Selleck
Jeff Settleman
Laurent Seugnet
Ben Seymour
Michael Shadlen
Reza Shadmehr
Ladan Shams
Jo Shaw
David Sheinberg
Michael Shen
Martha Shenton
Alan Sher
Toshiharu Shikanai
Ben-Zion Shilo
Alexander Shingleton
Ramesh Shivdasani
David Shore
Joshua Shulman
Susanne Shultz
Derek Sieburth
Neal Silverman
James Simmons
Lyle Simmons
M. Celeste Simon
Kai Simons
Michael Simons
Mikael Simons
Benjamin Simons
Laura Sim-Selley
L Sivilotti
William Skach
Marjan Slak Rupnik

James Slauch
Stephen Smith
Jake Snaddon
Jorge Soberon
Samuel Solomon
Ralf Sommer
Peter Somogyi
Holger Sondermann
Mike Speed
Hamish Spencer
Rebecca spencer
Michael Springer
Nelson Spruston
Ralf Stanewsky
John Stanton-Geddes
Thilo Stehle
David Stellwagen
Patrick Stephens
Philipp Sterzer
Charles Stevens
M. Henry Stevens
Jack Stilgoe
John Stingl
Ann Stock
Kate Storey
Lisa Stowers
Aaron Straight
Rob Striker
Michael Stumpf
Grace Stutzmann
Peter Sudbery
Greg Suh
Beth Sullivan
Christopher Summerfield
Ueli Suter
William Sutherland
Estienne Swart
Eörs Szathmáry
Dylan Taatjes
Takuya Takahashi
Yoshimi Takai
Marc Tatar
Jörg Tatzelt
Susan Taylor
Graham Taylor
Luis Teixeira
Carel ten Cate
Olivier Tenaillon
Christa Testerink
Ian Tetlow
Joshua Tewksbury
Randolph Thaman
Clotilde Thery
Craig Thomas
Kimberly Thompson
Gregor Thut
Paul Tiesinga
Andrew Tobin
Yukihide Tomari
Michael Tomasello
Nicholas Tonks
Sharon Tooze
Miguel Torres
Kazushige Touhara
Kay Trafford
Elizabeth Tran
Heather True-Krob
James Truman
Andreas Trumpp
Aaron Turkewitz
James Turner
Esther Turnhout
Mary Tyler
Francisco Úbeda
Masahiro Ueda
Markus Ullsperger
Helle Ulrich
Muriel Umbhauer
Kathleen Van Craenenbroeck
Russell Van Gelder
Marin van Heel
Francois Van Laethem
Pierre Vanderhaeghen
Wim Vanduffel
David Vaux
Fabrice Vavre
Gianluigi Veglia
Kartik Venkatachalam
Geerat Vermeij
Patrik Verstreken
Kevin Verstrepen
Jean-Philippe Vielle-Calzada
Monika Vig
Charles Vinson
Todd Vision
Marcel Visser
Edward Vogel
Hansjurgen Volkmer
Henrique von Gersdorff
Domagoj Vucic

Bernard W. Balleine
Jacques Wadiche
Tor Wager
Günter Wagner
Doris Wagner
Geoffrey Wahl
Mats Wahlgren
Loren Walensky
Graham Walker
Graeme Walker
David Wallach
John Wallingford
Peter Walter
Thomas Walz
Andrew Wang
Yanchang Wang
Erich Wanker
Andrew Ward
Stephen Ward
Joy Ward
Yuuki Watanabe
Nick Watkins
Felicity Watts
Wenyi Wei
Douglas Weibel
Robert Weinberg
William Weiss
Franz Weissing
Raymund Wellinger
John Werren
Stuart West
David Westaway
Chris Westlake
Johnathan Whetstine
James Whisstock
Rachel Whitaker
Eileen White
Malcolm Whiteway
David Whitney
Reed Wickner
David Wiest
John Wilbanks
Siouxsie Wiles
Claus Wilke
Jon Wilkins
Christopher Wilmers
Anna Wilson
Jeffrey Wilusz
Elliot Wimmer
Mark Winey
Joachim G. Wittbrodt
Kenneth Wolfe
Mariana Wolfner
Benjamin Wolozin
Melanie Woodin
Mark Wu
F. Gregory Wulczyn
Anton Wutz
Jun Xia
Mingyi Xie
Dajun Xing
Haoxing Xu
Daisuke Yamamoto
Keith Yamamoto
Yukiko Yamashita
Daiwen Yang
Lin Yang
Cindy Yang
George Yap
Alpha Yap
Kazufumi Yazaki
Bing Ye
Giles Yeo
Peng Yi
John Yin
Moira Yip
Satoshi Yoshida
Tamotsu Yoshimori
Lawrence Young
Dani Zamir
Kenneth Zaret
Lirong Zeng
Zhong-Yin Zhang
David Zhang
Mei Zhen
Zheng Zhou
Jia Zhou
Jian-Min Zhou
Ronghua Zhuge
Min Zhuo
Joshua Zimmerberg
Erik Zuiderweg
